# Long-Term Use of *Silybum marianum fruit extract* Contributes to Homeostasis in Acne-Prone Skin—A 12-Month Follow-Up International “Real Life” Cohort Study

**DOI:** 10.3390/jpm13010096

**Published:** 2022-12-31

**Authors:** Jean-Hilaire Saurat, Pascal Reygagne, Gwendal Josse, Zhorha Hamidou, Sophie Bianovici, Francoise Ramel, Elizabeth Durbise, Chiara Lovati, Eleonora Bellani, Dorota Bystrzanowska, Ewa Chlebus, Elzbieta Kowalska-Oledzka, Guillaume D’Auzers, Jimmy Le Digabel, Jerome Filiol, Christophe Lauze, Ariadna Ortiz-Brugues, Therese Nocera

**Affiliations:** 1 Department of Clinical Pharmacology and Toxicology, University of Geneva, 1205 Geneva, Switzerland; 2 Sabouraud Clinical Research Center, St Louis University Hospital, 75010 Paris, France; 3 Research & Development, Pierre Fabre Dermo-Cosmétique et Personal Care, 31035 Toulouse, France; 4 Independent Researcher, 75010 Paris, France; 5 Independent Researcher, 31000 Toulouse, France; 6 Independent Researcher, 20122 Milano, Italy; 7 Evimed Centrum Medyczne, Private Dermatologic Practice, JP Woronicza 16, 02-625 Warsaw, Poland; 8 LDermatology Department, Uczelnia Medyczna im. Marii Skłodowskiej Curie, 02-093 Warsaw, Poland; 9 Laboratoires Dermatologiques Avène, 81500 Lavaur, France; 10 Dermatology Department, University Hospital, 31000 Toulouse, France

**Keywords:** acne, microcomedones, lipid droplet proteins, sebum, phytotherapy, real-life study

## Abstract

Background: Homeostasis in the differentiation programme of sebaceous stem cells has been identified as a key step in comedogenesis and should be a target for acne-prone skin care. Objective: To report on a multicentre, year-long/real-life use study of a patented natural product containing *S. marianum fruit extract* proven to modulate molecular actors in the initial steps of comedogenesis. Methods: An open-label multicentric international study, with a 12 month follow-up, included 54 teenage and young adult subjects with mild to moderate facial acne. The study was aimed at reproducing a real-life use context. Results: Total lesion count mean was 88.3 at inclusion. There was a sustained, highly significant decrease over the months of clinical lesion counts (45.6% improvement after 6 months and 59.6% at 12 months) and on other efficacy markers, associated with a significant decrease in global microcomedone quantity on cyanoacrylate superficial skin surface biopsies. Importantly, the study protocol allowed the dermatologist to prescribe, if needed as in real life, any of the acne drugs registered in the acne guidelines. The exposure to these acne drugs during the whole year was calculated as a percentage of *S. marianum fruit extract*/352 days of use and happened to be very limited at less than 4%, which indicates a marginal contribution to the sustained clinical improvement. (Oral and local acne treatments: Lymecycline 1.46%; Doxycycline 0.24%; Adapalene 0.16% or gel association with Benzoyl peroxide 1.17%; Clindamycin 0.04%; Benzoyl peroxide 1.5%; Erythromycin 0.75%). The tolerance with daily *S. marianum fruit extract* long-term use was good. Limitations: The association with routine prescription acne drugs when needed, even if limited, does not allow a full evaluation of the intrinsic quantitative efficacy of *S. marianum fruit extract* in lesion reduction. Conclusion: This open, real-life, year-long multicentre study confirms a previous 48-week proof of concept study and qualifies the use of *S. marianum fruit extract* as a “field-dermo cosmetic” contributing to homeostasis of acne-prone skin in association with acne drugs.

## 1. Introduction

In acne-prone skin, besides visible acne lesions, normal-appearing facial skin is stuffed with infra-clinical abnormalities called microcomedones; they are the first step for all future clinical lesions and locus minoris resistentiae for C. acne growth [1,2].

Microcomedone formation corresponds to a switch in the differentiation programme of sebaceous stem cells located in the epithelium of the isthmus: in homeostasis, these stem cells are able, through their lineage, to generate both the sebaceous gland itself (sebaceous differentiation) and the epithelia of the sebaceous duct and isthmus (epithelial duct differentiation). Loss of homeostasis in acne-prone skin results in the expansion of the epithelial one, which is the root of the microcomedone [3,4,5,6,7].

Maintaining homeostasis in this system has appeared as a new goal in acne patients [3]. Since sebaceous stem cells are located in the epithelium of the isthmus, closer to the surface, they are more accessible to topicals than the sebaceous gland itself. This gives more credit to topical delivery for this target. Molecular pathways potentially involved in the lineage determination of sebaceous stem cells and the comedone-switch have been identified [3,4]. *S. marianum fruit extract (SMFE)* was identified as the first topical extract able to interfere with this comedone-switch mechanism. *SMFE* protects sebocytes exposed in vitro to a strong comedogen TCDD and modulates the expression of Keratins 75 and 79 [6] and lipid droplet proteins such as Plin2 and CIDEA which are produced by sebaceous stem cell lineage [7].

In a proof of concept study on 23 subjects, the use of *SMFE* for 48 weeks was associated with an initial decrease in the microcomedone index (as shown in repeated skin surface biopsies) and maintenance of a low microcomedone index over the months. This lowering of the microcomedone index correlated with a similar decrease in clinical lesions [6]. 

Since 2019, a product based on SMFE has been available worldwide as a “field-cosmetic” to maintain homeostasis in the acne-prone skin of teenagers and young adults and is currently used for long periods of time. Therefore, information on long-term tolerability, as well as compatibility, in association with acne prescription drugs is necessary. The present 12-month multicentre international study was aimed at reproducing the single centre proof of concept study in a real-life use context.

## 2. Methods

This study was an open-label multicentric international study, with a 12 month follow-up, on 54 teenage and young adult subjects with mild to moderate acne of the face, aged between 12 and 35 years. The study protocol was performed in the spirit of Good Clinical Practice and obtained written informed consent, in accordance with local regulations, from volunteers, parents, or guardians. 

The study was registered with Clinical Trials.gov ID: NCT04301063.

Before the subject’s inclusion, a wash-out period for acne treatments was required (oral intake of isotretinoin within 6 months; hormonal contraception or hormonal anti-acne treatment established or modified within 3 months; any oral or topical anti-acne treatment taken/applied on the face within 1 month). A wash-out period was also respected for acne cosmetic products (facial cleansing or skin-care products containing anti-acne or keratolytic ingredients applied within 15 days before the inclusion or planned to be applied during the study).Any modifications to a subject’s hygiene regimen had to occur within the 15-day period prior to the inclusion date.

Clinical and instrumental evaluations were performed at baseline (visit 1) and after the month 1 (visit 2), month 4 (visit 3), month 6 (visit 4), month 9 (visit 5), and the month 12 end of the study (visit 6) follow-ups.

Clinical assessments included: a lesion count according to the Lucky method [8], a Global Evaluation of Acne by an investigator (GEA 6-point severity scale ranged from 0 to 5) [9], a Subject’s Global Assessment (7-point dynamic severity scale from −3 to 3), and local cutaneous tolerance. Any information about acne relapses/flare-ups that occurred during the follow-up period was collected by dermatologists. Any relapses defined as GEA ≥ 3 (moderate severity) and any acne oral and/or topical drug therapy required were judged by an investigator.

To reproduce a real-life context, the prescription of any anti-acne drugs was left at the discretion of the investigator, depending on the acne severity, according to the international guidelines’ recommendation [10] and carefully reported in the patients’ files.

The Cardiff Acne Disability Index questionnaire (CADI) [11] was completed by each subject at baseline, after 6 months, and at the end of the study at 12 months. It consisted of 5 questions regarding the impact of acne on the subject’s quality of life, and each response was on a 4-point scale (global score was 0 to 20).

Cyanoacrylate Skin surface stripping (CSSS) of the forehead was performed according to Pierard et al. [12] and described by Fontao et al. [6] for microcomedone analysis. The procedures were in accordance with Helsinki rules, with written consent from all the volunteers and after establishing the absence of registered opposition to the use of tissue samples according to the guidelines of local Bioethics Law. CSSS was done at each of the 6 study visits by the investigators in French centres. In the other centres (Italy and Poland), these samplings were performed at baseline and at visits 4 and 6 (after 6 and 12 months, respectively).

Image acquisition was performed directly on CSSS samples, and the microcomedone density and surface were computed using dedicated image analysis software [2].

### Tested Product Containing SMFE

We had identified the comedolytic properties of a specific *SMFE* in the context of a patient-based, data-oriented programme for fighting xenobiotic comedogens (European patent EP3478309) [6]. A topical skin-care preparation based on patented *SMFE (Cleanance Comedomed Anti-Blemishes Concentrate^®^*, *Laboratoire Dermatologique Eau Thermale Avène)* was applied twice a day during the whole study period of 52 weeks.

Illustrative face photos were also performed at different visits using normal light.

A specific mobile application (Clear Skin Partner^®^) was developed to improve the patient commitment during the study, used as a self-diary for the compliance evaluation and to improve investigator follow-up and the patient’s interaction between visits. 

Due to the exploratory nature of the study, all statistical results were expressed in a descriptive form. Analyses were performed on the FAS (Full Analysis Set). In cases where more than 15% of subjects had major protocol deviations (or other sources of bias), the analyses would have been repeated on a PP (Per Protocol) set. However, it was decided to conduct inferential post hoc analyses to reinforce the observed results. For the counting of acne lesions, CADI, and CSSS, an analysis of variance with repeated measures was performed, with “visit” as the fixed effect and “subject” as the random effect. For the GEA, the Wilcoxon signed-rank test was performed on changes that occurred after the baseline start date.

## 3. Results

**Figure 1:**
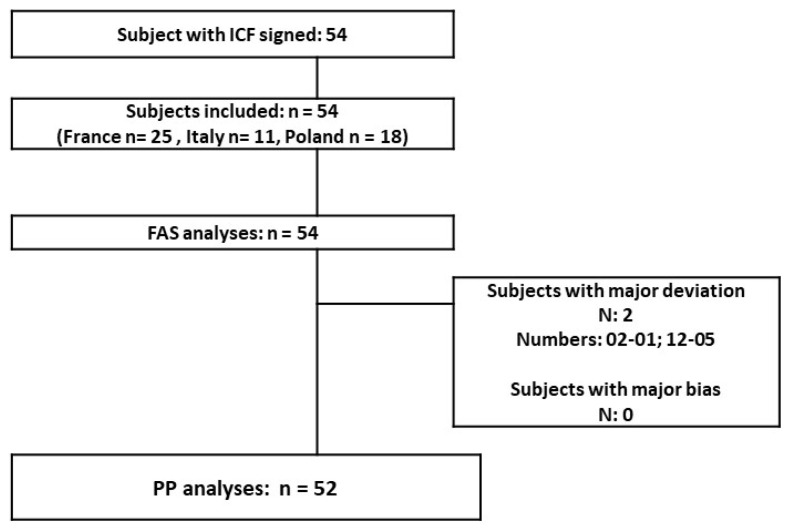
Study population.

Study population: 54 teenagers and young adults were included (12 to 34 years old; mean age 19.5). Most of the subjects were female (79.63%) and the teenage population accounted for 42.59% of the total. A great majority of the skin phototypes (I to IV) were type II–III. At the baseline, the population was globally homogenous (mild to moderate GEA severity score); only the Polish population had a slightly lower acne severity with 3/18 included subjects having an “almost clear” rating. The disposition of subjects in the different populations presented (Figure 1): 54 subjects are in the FAS population and 52 are in the PP population (2 major protocol deviations in relation with forbidden treatments before inclusion and during the study). There was no associated acne drug treatment at baseline in the included population. The COVID-19 lockdown period was concomitant to the 9- and 12-month follow-ups (visits 5 and 6).

### 3.1. Lesion Counts Evaluation

**Figure 2:**
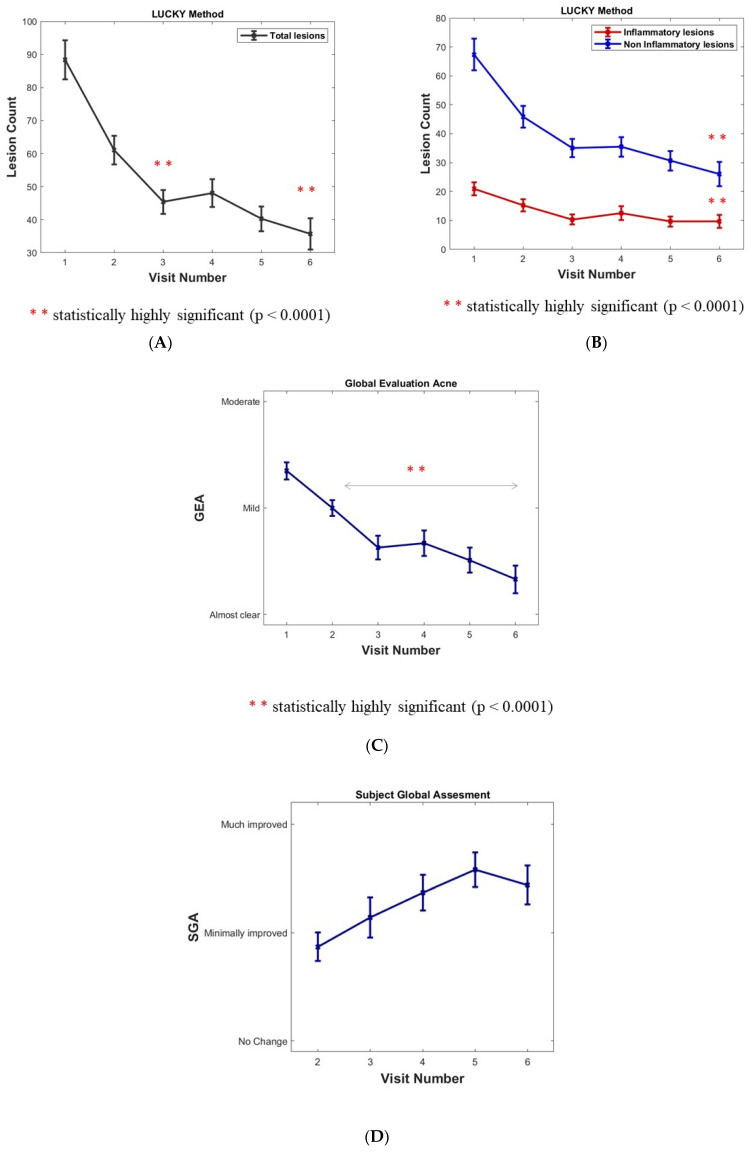
(**A**) Assessment of acne in cohort population: Acne lesion count. (**B**) Inflammatory and Non-inflammatory acne lesions (open and closed comedones). Results are expressed as means ± SD; a sustained, statistically significant decrease versus baseline after 6 months (Visit 4) and 12 months (Visit 6) for lesion count (*p* < 0.0001). (**C**) Acne assessment using the investigator Global Evaluation of Acne (GEA). Results are expressed as means ± SD. *p* value < 0.0001 for ∆(Vx-V1) x = 2 to 6. (**D**) Subject’s Global Assessment (SGA) of acne improvement after the 6- and 12-month follow-ups.

The total lesion count mean was 88.3 at inclusion. There was a sustained and highly significant decrease in the Lucky score over the months (minus 45.6% and 59.6% at 6 and 12 months, respectively, *p* value < 0.0001 inferential analyses) (Figure 2A).

Non-inflammatory lesions (NIL open and closed comedones) represented 76% of total lesions with a mean of 67.4 NIL at inclusion. A significant decrease and maintenance over the months were observed (minus 61.4% at 12 months, *p* value < 0.0001 inferential analyses). A plateau at 4 and 6 months corresponded to the summer period (Figure 2B).

Inflammatory lesions (per protocol without nodules) represented 24% of total lesions with a mean of 20 lesions at inclusion. A significant decrease and maintenance over the months (minus 54% at 12 months, *p* value < 0.0001 inferential analyses). A plateau at 4 and 6 months corresponded to the summer period (Figure 2B).

### 3.2. Global Evaluation of Acne GEA

The global evaluation of acne (GEA) method was chosen rather than the IGA because it better captures variations in lesion counts in mild to moderate acne which was the per protocol target of the trial [9]. A significant decrease and maintenance were observed over the months (*p* value < 0.0001 for ∆(Vx-V1) x = 2 to 6). During the plateau corresponding to the summer period at 4 and 6 months, the GEA decrease remained significant (Figure 2C). The percentage of “clear and almost clear” reports rose significantly over the months from 0 and 5.5% to 4 and 40.8%, and then to 21.5 and 33.3% at 6 and 12 months, respectively (*p* value < 0.0001 signed-rank test between inclusion and both months 6 and 12).

A combined rate of the evolution of the GEA scores was compared with the baseline throughout the study period (% of improved, unchanged, worsened subjects): an increase in improved subjects of 38.9% at 1 month; 57.1% at 6 months; and 72.5% at 12 months of the study visit were observed. A low, background worsening of the GEA over a 12-month period could possibly be in relation to acne flare-ups. 

At 9 and 12 months, respectively, visit 5 and visit 6 corresponded to a period of COVID-19 lockdown.

### 3.3. Tolerance Compliance and Safety 

After 12 months of product use, global tolerance was judged by the investigator as “excellent” for 98% of subjects. Few withdrawals (6) occurred over the 12 months, and only one was due to product intolerance (dryness, itching). The evolution of local tolerance improved over time. Product compliance was very good.

### 3.4. Acne Flare-ups and Prescription Drugs Use

**Figure 3:**
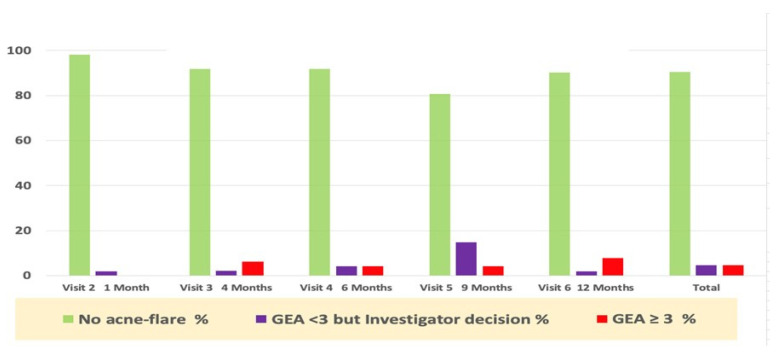
Percentage of subjects with acne flare-ups and prescription drug use during the study.

Acne worsening (flare-up), defined as “GEA ≥ 3 and treatment required” was reported in eight subjects during the whole study period. Only six subjects required per protocol antibiotic treatment (3 oral and 3 topical were was prescribed). Only one subject was treated with an oral antibiotic, despite a GEA = 2. The majority of first flare-ups (6/8) occurred during the first six months of the study period (Figure 3).

In addition to antibiotics, the study protocol allowed the dermatologist to prescribe, if perceived as necessary outside of an acne flare-up as defined in the study protocol, any of the acne drugs registered in the acne guidelines, as in real life. 

Table 1 recapitulates those prescription drugs used during the whole year of the trial. The subject’s exposure to these oral and topical drugs was calculated as a percentage of *SMFE*/days of use over one year. These data show that exposure to these drugs was very limited, and their contribution to clinical improvement was likely marginal. The average delay of the first relapse was within 129 days of the study follow-up period. Nevertheless, this confirms the compatibility of these drugs with daily *SMFE* long-term use as suggested by the pre-marketing *SMFE* data [7].

### 3.5. Subject Global Assessment

The subject’s global assessment score (SGA) at the 6- and 12-month study visit: since the first month, 74% of the subjects declared their acne was “minimally, much, very much improved”. The SGA scores are 77.5% at 6 months and 81.2% after 12 months of tested product use. Interestingly 89.6% of the subjects also rated the skin moisturizing effect of the product (Visit 3 after the 4 month follow-up).

### 3.6. Cardiff Acne Disability Index (CADI)

**Figure 4:**
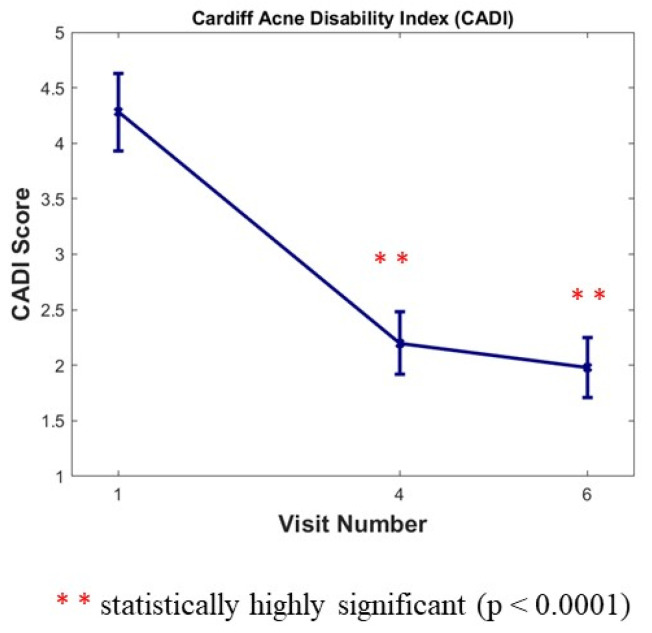
Cardiff Acne Disability Index (CADI) score 0 to 20. Results are expressed as means ± SD; a large statistically significant decrease versus baseline after 6 months (Visit 4) and after 12 months (Visit 6) for this index (*p* < 0.0001).

The CADI was completed by subjects three times during the study (at Baseline, and at month 6 and month 12). We noticed a large statistically significant decrease versus baseline of 48.60% after 6 months (Visit 4) and of 53.74% after 12 months (Visit 6) for this index (*p* < 0.0001) (Figure 4).

### 3.7. Cyanoacrylate Superficial Skin Surface Biopsy (CSSS)—Microcomedones Counts

**Figure 5:**
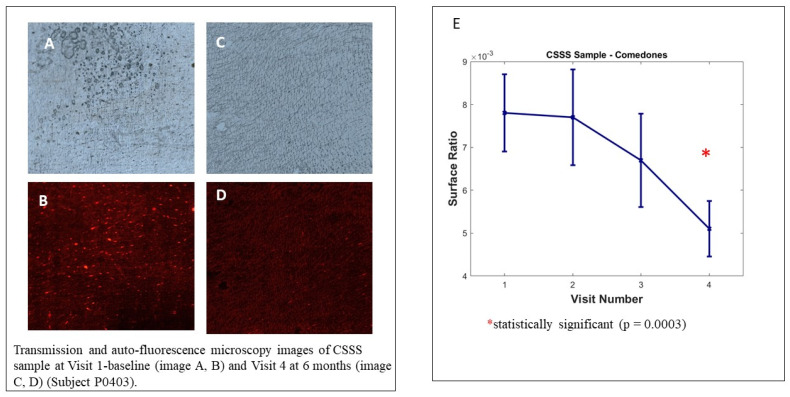
Cyanoacrylate superficial skin surface (CSSS) biopsy analysis. Transmission and auto-fluorescence microscopy images of CSSS sample at Visit 1 baseline (image **A**,**B**) and Visit 4 at 6 months (image **C**,**D**) (Subject P0403). Image (**E**) Sample analysed with Mixed Model for Repeated Measures (MMRM), using visit and country as fixed factor and subject as random factor.

We observed no significant decrease in microcomedone density on the analysed samples; however, the intra-group analysis by visit, illustrated in Figure 5, shows the decrease in the surface ratio of microcomedones between the baseline visit and visit 4 (6 months). This evolution represents the decrease in overall microcomedone quantity on CSSS and is highly significant (*p* = 0.0003). The poor samples collected at visit 6 (12 months) did not allow us to reach statistically significant results because the COVID-19 pandemic strongly interfered in the study conduct.

### 3.8. Mobile Application (Clear Skin Partner^®^)

A specific mobile application was developed for our long-term study: to improve patients’ commitment during the study; to be used as a self-diary for the compliance evaluation and collection of cutaneous adverse events; and finally, to improve investigator–patient follow-ups and interaction between visits and in the reminder of visits.

Connexions and screen views (total of 81,564; approximately 1500/volunteer) during the study were in coherence with clinical centres (France, Poland, and Italy). The average mobile app visit time was 2 min and 3 s. In evaluating the mobile app, 77.8% of enroled volunteers declared that they were “very satisfied” (33.3% of them) and “satisfied” (44.4%) with the mobile app”.

## 4. Discussion

This open, real-life, year-long multicentre study confirms a previous 48-week proof of concept study [6]. It is not usual for a dermocosmetic product to be submitted to such a clinical programme. We felt this was mandatory to better validate the clinical relevance of the homeostatic effect of *SMFE* observed in the previous stages of its profiling.

The homeostatic effect of *SMFE* in acne-prone skin likely results from a combination of factors: 

Although a reduction of sebum excretion after 8 weeks in a previous clinical trial including 56 patients (cited in Sorg O. et al. [7]) was significant at minus 38%, it was still lower than the typically required level for clinical relevance in acne.

A decrease in the proportion of free fatty acids in the sebum was observed after SMFE use in two trials^7^. For decades, free fatty acids have been considered as comedogenic, and their increase contributes to a loss of homeostasis.

*SMFE* was the first product found to increase the expression of proteins in the skin whose function is to prevent the formation of free fatty acids [7]. These lipid droplet proteins may be key contributors in sebum homeostasis.

*SMFE* also increases the expression of K75 and K79 in the skin, two keratins produced by the progeny of Lrig1+ sebocytes stem cells, and specifically expressed in infundibulum-isthmus-sebaceous duct epithelia [6]. This may be either just a marker of restored homeostasis or it may have functional significance; some properties of these keratins may well contribute to the special function of the isthmus epithelium, and their decrease may contribute to microcomedone formation [5].

This study qualifies the use of *SMFE* as a “*field dermocosmetic”* for maintaining homeostasis of acne-prone skin. The concept of “*field dermocosmetic*” captures the fact that, besides visible acne lesions, normal-appearing facial skin (*the field*) is stuffed with infra-clinical abnormalities indicating a loss of homeostasis and corresponding both to embryos of future clinical lesions and locus minoris resistentiae for C. acne growth [1,2]. Therefore, a dermocosmetic with an acne-prone skin label should, at least, be non-comedogenic (non-aggravating; no loss of homeostasis). At best, it should contribute to reinstall and maintain homeostasis, which would qualify it as “*field dermocosmetic acne-prone skin*”. Moreover, since treatment adherence is key to improve efficacy, field dermocosmetics in acne-prone skin need to have moisturizing properties in order to balance the xerosis and irritation due to topical acne treatments [13].

It is not unlikely that some of the many dermocosmetics on the market dedicated to acne-prone skin may modulate these novel molecular players in acne-prone skin homeostasis. This may open the door for a complementary profiling of active non-drug dermocosmetic care to the advantage of the consumers.

## Figures and Tables

**Table 1:** Oral and topical drugs used (days) during the 12-months follow-up period.

Oral drugs	Number of days of use during 12 months						
	Doxycycline 100			Limecyclin 300			
Total Days on	43			265			
Nb Patients on	2			2			
% of Total Days on	0.24			1.46			
Topical drugs	Number of days of use during 12 months						
	Adapalene	Adapalen + PBO gel 0.3%	BPO	Clinda BPO	Erythromycin	Clindamycin	Aknemycin (Erythromycin)
Total Days on	30	213	271	3	72	8	66
Nb Patients on	1	3	2	2	2	1	1
% of Total Days on	0.16	1.17	1.5	0.016	0.39	0.04	0.36
